# Supplementary material for: Nationwide geospatial analysis of county racial and ethnic composition and public drinking water arsenic and uranium
Source: Nat Commun. 2022 Dec 3;13:7461. doi: 10.1038/s41467-022-35185-6 (PMC9718774; doi:10.1038/s41467-022-35185-6)
Supplement: Supplementary file 1 — Supplementary Information [file 41467_2022_35185_MOESM1_ESM.pdf]

Nationwide geospatial analysis of county-level racial/ethnic composition and public drinking water arsenic and uranium.

Pg.3	**Supplementary Fig. 1.** Nationwide spatial distribution of county-level community water system (CWS) metal concentrations and local indicators of spatial associations (LISA) clusters for barium and selenium.
Pg.4	**Supplementary Figure 2.** Directed acyclic graph for the county-level association between racial/ethnic composition and public drinking water metal concentrations.
Pg.5	**Supplementary Table 1.** Sensitivity analyses of geometric mean ratios (95% CI) of county-level community water system (CWS) metal concentration estimates (µg/L) per increases in the proportion of non-Hispanic African American, American Indian, Hispanic, and non-Hispanic White residents, from spatial lag regression models, assessing A) increases in the proportion of residents in the specified racial/ethnic group of 0.6, B) increases in the proportion of residents in the specified racial/ethnic group corresponding to the interquartile range (IQR), and C) per unit increase in the proportion of residents in the specified racial/ethnic group, restricted to counties in the western US.
Pg.6	**Supplementary Table 2.** County-level community water system (CWS) arsenic and uranium concentrations per a 10 percent higher proportion of all residents not categorized as non-Hispanic White, including non-Hispanic Black, American Indian/Alaskan Native, Hispanic/Latino, from spatial lag regression models
Pg.7	**Supplementary Table 3.** County-level community water system (CWS) barium and selenium concentrations per a 10 percent higher proportion of non-Hispanic Black, American Indian/Alaskan Native, Hispanic/Latino, and non-Hispanic White residents, from spatial lag regression models
Pg.8	**Supplementary Fig. 3.** County-level R^2 values from geographically weighted regression models assessing the association between a 10 percent increase in the proportion of county population categorized as Hispanic, American Indian or Alaskan Native, non-Hispanic African American, and non-Hispanic White and geometric mean county-level community water system (CWS) arsenic.
Pg.9	**Supplementary Fig. 4.** County-level R^2 values from geographically weighted regression models assessing the association between a 10 percent increase in the proportion of county population categorized as Hispanic, American Indian or Alaskan Native, non-Hispanic African American, and non-Hispanic White and geometric mean county-level community water system (CWS) uranium.
Pg.10	**Supplementary Fig. 5.** County-level R^2 values from geographically weighted regression models assessing the association between a 10 percent increase in the proportion of county population categorized as Hispanic, American Indian or Alaskan Native, and non-Hispanic African American, and geometric mean county-level community water system (CWS) barium and selenium.
Pg.11	**Supplementary Fig. 6.** Direction and magnitude of the geometric mean ratio (GMR) of county-level community water system (CWS) barium concentrations per 10 percent higher county proportion of non-Hispanic Black, American Indian/Alaskan Native, Hispanic/Latino, and non-Hispanic White residents from geographically weighted regression models.
Pg.12	**Supplementary Fig. 7.** Direction and magnitude of the geometric mean ratio (GMR) of county-level community water system (CWS) selenium concentrations per 10 percent higher county proportion of non-Hispanic Black, American Indian/Alaskan Native, Hispanic/Latino, and non-Hispanic White residents from geographically weighted regression models.
Pg.13	**Supplementary Table 4**. County-level mean estimated community water system (CWS) metal concentrations and sociodemographic characteristics for all counties excluded from the analysis (counties with < 100 residents of each racial/ethnic group).
Pg.14	**Supplementary Table 5.** Description and source of county-level variables considered and utilized in the current analysis
Pg.16	**Supplementary Table 6.** Diagnostics for spatial lag and spatial error models from ordinary least squares regression models assessing the association between racial/ethnic composition and community water system metal concentration estimates.

Pg.17	**Supplementary Fig. 8. Photocopy safe version for main manuscript Figures 2 and 3.** Direction and magnitude of the geometric mean ratio (GMR) of county-level community water system (CWS) arsenic and uranium concentrations per 10 percent higher county proportion of non-Hispanic Black, American Indian/Alaskan Native, Hispanic/Latino, and non-Hispanic White residents from geographically weighted regression models

Supplementary Fig. 1. **Nationwide spatial distribution of county-level community water system (CWS) metal concentrations and local indicators of spatial associations (LISA) clusters for barium (a) and selenium(b).** County-level CWS metal concentrations were originally developed and described by Nigra et al. 2020 and Ravalli et al. 2022 and are categorized and colored by quintile in red scale. High-High LISA clusters are shown in red, and Low-Low LISA clusters are shown in blue. Counties overlaid with gray hatch marks are missing CWS metal concentration estimates.

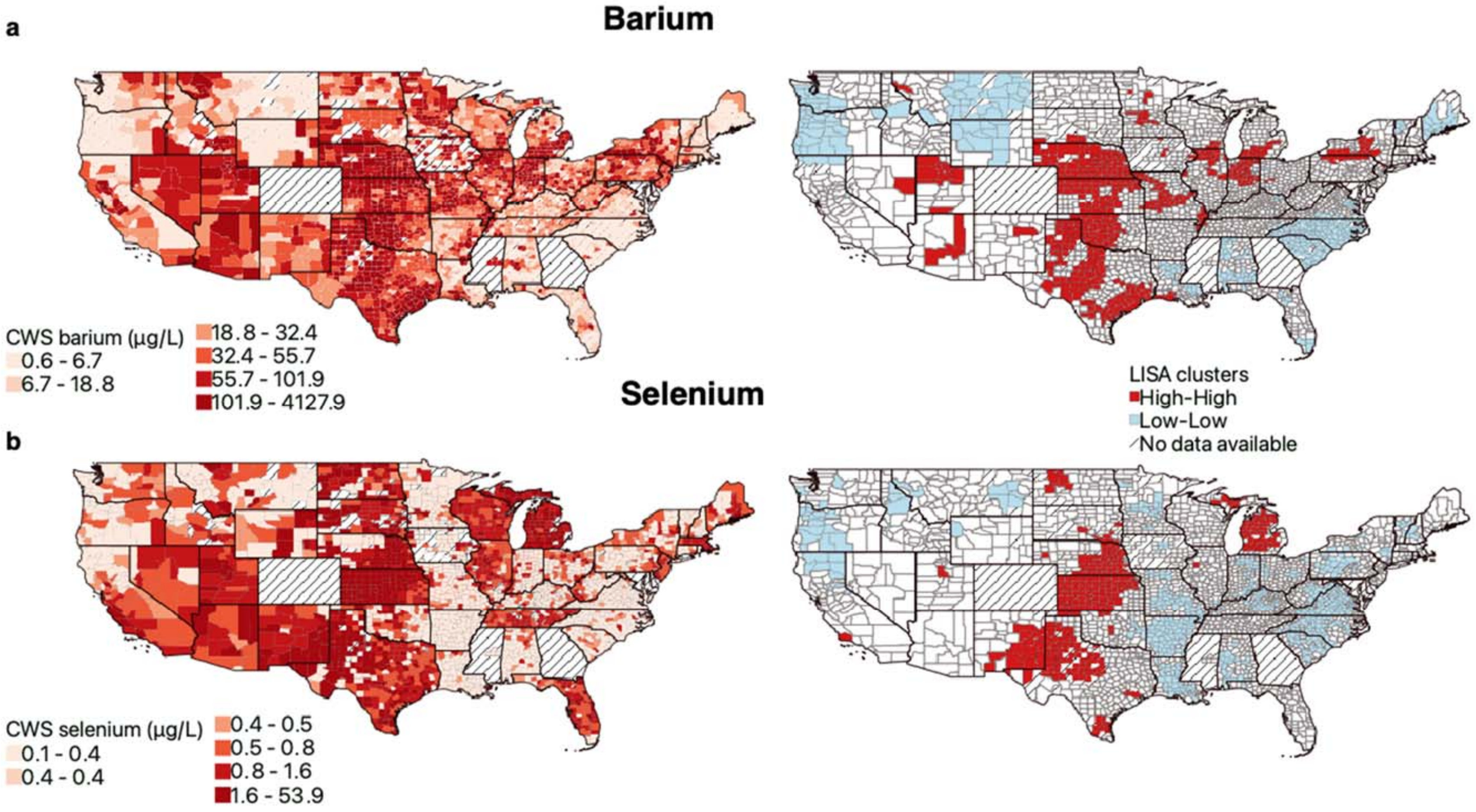

Supplementary Fig. 2**. Conceptual diagram for the county-level association between racial/ethnic composition and public drinking water metal concentrations**. Created at www.dagitty.net. The minimal sufficient adjustment set for estimating the direct effect of racial/ethnic composition on public water metal concentrations is: percent of other racial/ethnic groups, the percent of public water from groundwater supplies, population density, the percent of residents with a high school diploma, and median household income. Although we primarily treat median household income and the percent of adults with a high school diploma as confounders of the association between county racial/ethnic composition and public drinking water metal concentrations, these variables may also act as mediators of the association.

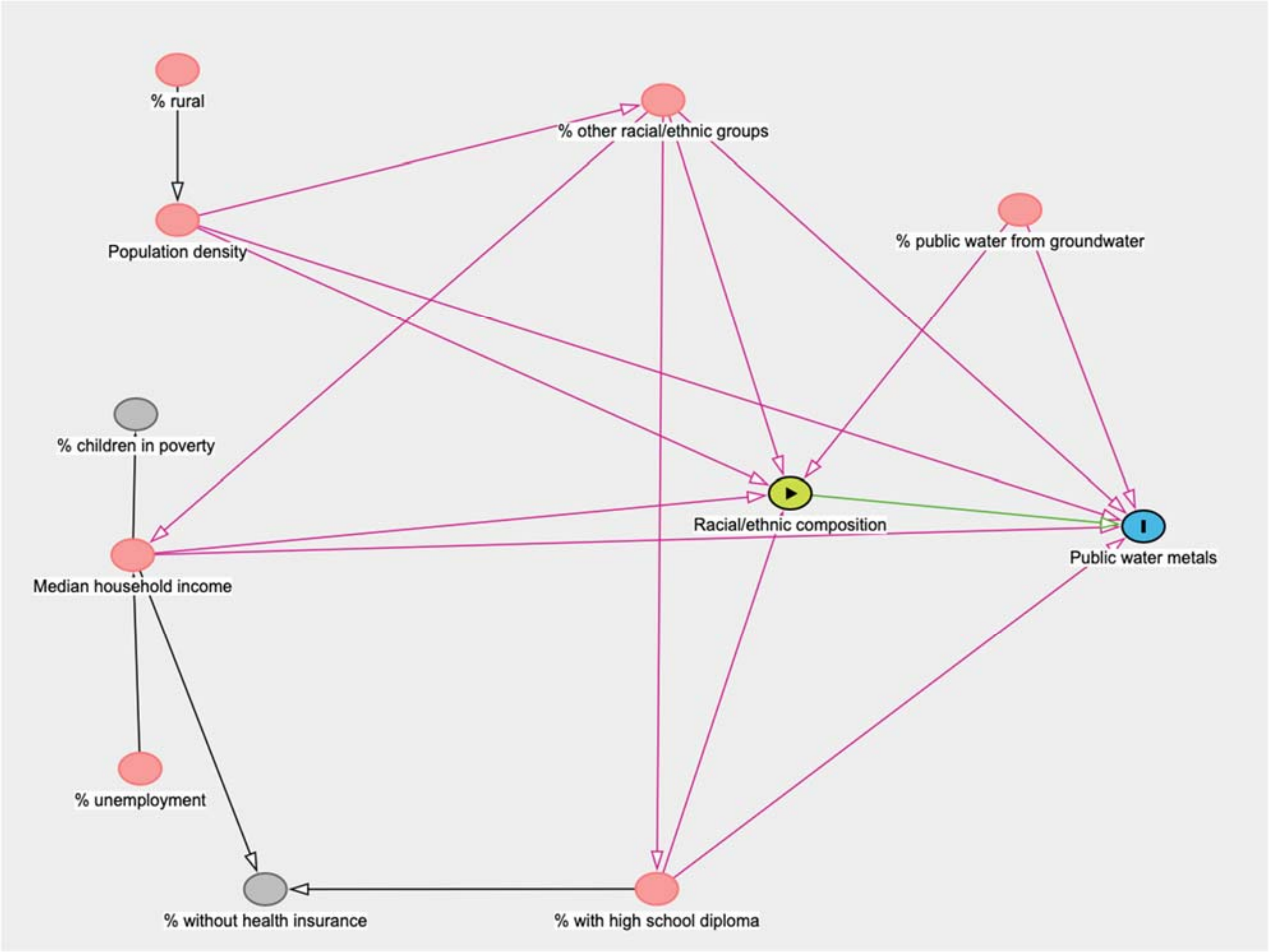

Supplementary Table 1**. Sensitivity analyses of geometric mean ratios (95% CI) of county-level community water system (CWS) metal concentration estimates (µg/L) per higher proportion of non-Hispanic Black, American Indian/Alaskan Native, Hispanic/Latino, and non-Hispanic White residents, from spatial lag regression models, assessing A) 60% higher proportion of residents in the specified racial/ethnic group, B) higher proportions of residents in the specified racial/ethnic group corresponding to the interquartile range (IQR), and C) per 10% higher proportion of residents in the specified racial/ethnic group, restricted to counties in the western US.** Spatial autocorrelation was modeled in Lagrange models with autoregressive correlation structure. County-level CWS arsenic, barium, selenium, and uranium were natural log-transformed for analysis. Models were adjusted for population density, the percent of public water sources from groundwater supplies, median household income, and the percent of adults with a high school diploma (Model 3). Coefficients for the spatial lag term were significant in all models.

		**A. Per 60% higher proportion of residents of the specified racial/ethnic group**	**B. Per higher proportion of residents of the specified racial/ethnic group corresponding to the IQR**	**IQR value**		**C. Per 10% higher proportion of residents of the specified racial/ethnic group, restricted to counties in the western US**
	**N**	**GMR (95% CI)**	**GMR (95% CI)**		**N**	**GMR (95% CI)**
Hispanic/Latino						
Arsenic	2341	1.40 (1.26, 1.56)	1.04(1.03, 1.06)	0.08	828	1.03 (1.00, 1.05)
Uranium	170	2.62 (2.09, 3.28)	1.13 (1.10, 1.17)		475	1.11 (1.06, 1.17)
non-Hispanic Black						
Arsenic	1848	0.62 (0.55, 0.71)	0.92 (0.90, 0.94)	0.10	526	0.89 (0.78, 1.01)
Uranium	832	0.57 (0.44, 0.75)	0.91 (0.87, 0.95)		296	1.13 (0.82,1.55)
American Indian/Alaskan Native						
Arsenic	1522	1.55 (1.23, 1.96)	1.01 (1.00, 1.01)	0.01	640	1.02 (0.97, 1.08)
Uranium	778	1.17 (0.82, 1.66)	1.00 (1.00, 1.01)		391	0.94 (0.88, 1.02)
non-Hispanic White						
Arsenic	2585	0.94 (0.86, 1.01)	0.97 (0.94, 1.01)	0.24	939	0.99 (0.96, 1.01)
Uranium	1174	0.71 (0.60, 0.84)	0.87 (0.81, 0.93)		548	0.94 (0.90, 0.98)

Supplementary Table 2**. Geometric mean ratios (95% CI) of county-level community water system (CWS) arsenic and uranium concentrations per a 10 percent higher proportion of all residents not categorized as non-Hispanic White, including non-Hispanic Black, American Indian/Alaskan Native, Hispanic/Latino add the other Census groups here, from spatial lag regression models.** Spatial autocorrelation was modeled in Lagrange models with autoregressive correlation structure. County-level CWS arsenic and uranium were natural log-transformed for analysis. Model 1 adjusts for population density, the percent of public water sources from groundwater supplies, median household income and the percent of adults with a high school diploma. Model 2 further adjusts for the racial/ethnic composition of other racial/ethnic groups, except non-Hispanic White (leave-one-out model). Coefficients for the spatial lag term were significant in all models

	**% Non-Hispanic White**		**All participants not categorized as non-Hispanic White**	
	GMR	% Change	GMR	%Change
**Arsenic**				
N	2,585		2511	
Model 1	0.99 (0.98, 1.00)	-1% (-2, 0)	1.01 (0.99, 1.03)	1% (-1, 3)
Model 2				
**Uranium**				
N	1,174		1,148	
Model 1	0.95 (0.92, 0.97)	-5% (-8, -3)	1.10 (1.06, 1.14)	10% (6, 14)
Model 2				

Supplementary Table 3**. Geometric mean ratios (95% CI) of county-level community water system (CWS) barium and selenium concentrations per a 10 percent higher proportion of non-Hispanic Black, American Indian/Alaskan Native, Hispanic/Latino, and non-Hispanic White residents, from spatial lag regression models.** Spatial autocorrelation was modeled in Lagrange models with autoregressive correlation structure. County-level CWS barium and selenium were natural log-transformed for analysis. Model 1 adjusts for population density, the percent of public water sources from groundwater supplies, median household income and the percent of adults with a high school diploma. Model 2 further adjusts for the racial/ethnic composition of other racial/ethnic groups, except non-Hispanic White (leave-one-out model). Coefficients for the spatial lag term were significant in all models. [a]Sensitivity analyses not adjusting for population density yielded similar findings.

	**% Non-Hispanic Black**		**% American Indian/ Alaskan Native[a]**		**% Hispanic/Latino**		**% Non-Hispanic White**		**% All participants not categorized as non-Hispanic White**	
	GMR	% Change	GMR	% Change	GMR	% Change	GMR	% Change	GMR	%Change
**Selenium**										
N	1,848		1,522		2,341		2,555		2,512	
Model 1	0.96 (0.94, 0.98)	-4% (-6, -2)	1.03 (1.00, 1.07)	3% (0, 7)	1.05 (1.03, 1.07)	5% (3, 7)	0.99 (0.97, 1.00)	-1% (-3, 0)	1.02 (1.01, 1.03)	2% (1, 3)
Model 2	0.97 (0.95, 0.99)	-3% (-5, -1)	1.03 (1.00, 1.07)	3% (1, 7)	1.05 (1.03, 1.07)	5% (3, 7)				
**Barium**										
N	1848		1522		2,341		2,555		2,512	
Model 1	0.86 (0.82, 0.89)	-14% (-18, -11)	1.08 (1.01, 1.15)	8% (1, 15)	1.05 (1.02, 1.08)	5% (2, 8)	1.01 (0.98, 1.03)	1% (-2, 3)	0.98 (0.95, 1.01)	-2% (-5, 1)
Model 2	0.86 (0.83, 0.90)	-14% (-17, -10)	1.05 (0.98, 1.12)	5% (-2, 12)	1.04 (1.01, 1.08)	4% (1, 8)				

Supplementary Fig. 3. **County-level R^2 values from geographically weighted regression models assessing the association between a 10 percent higher proportion of county population categorized as Hispanic/Latino, American Indian/Alaskan Native, non-Hispanic Black, and non-Hispanic White residents and geometric mean county-level community water system (CWS) and arsenic.** High R^2 values are shown in darker colors and indicate a higher goodness-of-fit. Models were adjusted for population density, the percent of public drinking water supplied from groundwater sources, median household income, and the percent of residents with a high school diploma. Counties with missing data are shown with light gray hatch marks. The number of counties included in the geographically weighted regression analysis for each racial/ethnic group were: Non-Hispanic Black (n= 1,848), American Indian/Alaskan Native (n= 1,522), Hispanic/Latino (n= 2,341), and Non-Hispanic White (n = 2,585)

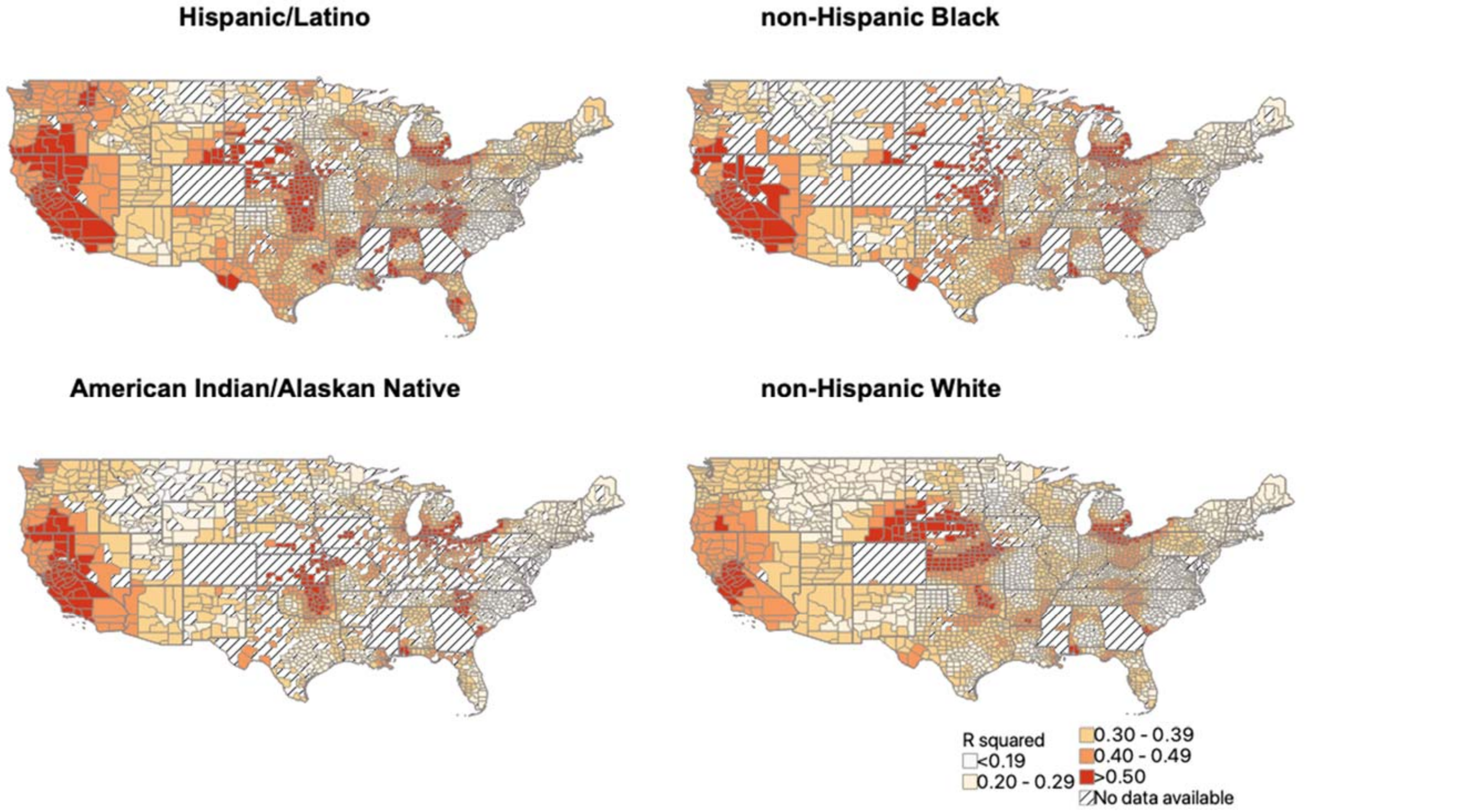

Supplementary Fig. 4**. County-level R^2 values from geographically weighted regression models assessing the association between a 10 percent higher proportion of county population categorized as Hispanic/Latino, American Indian/Alaskan Native, non-Hispanic Black, and non-Hispanic White residents and geometric mean county-level community water system (CWS) and uranium.** High R^2 values are shown in darker colors and indicate a higher goodness-of-fit. Models were adjusted for population density, the percent of public drinking water supplied from groundwater sources, median household income, and the percent of residents with a high school diploma. Counties with missing data are shown with light gray hatch marks.The number of counties included in the geographically weighted regression analysis for each racial/ethnic group were: Non-Hispanic Black (n= 832), American Indian/Alaskan Native (n= 778), Hispanic/Latino (n= 1,170), and Non-Hispanic White (n = 1,174)

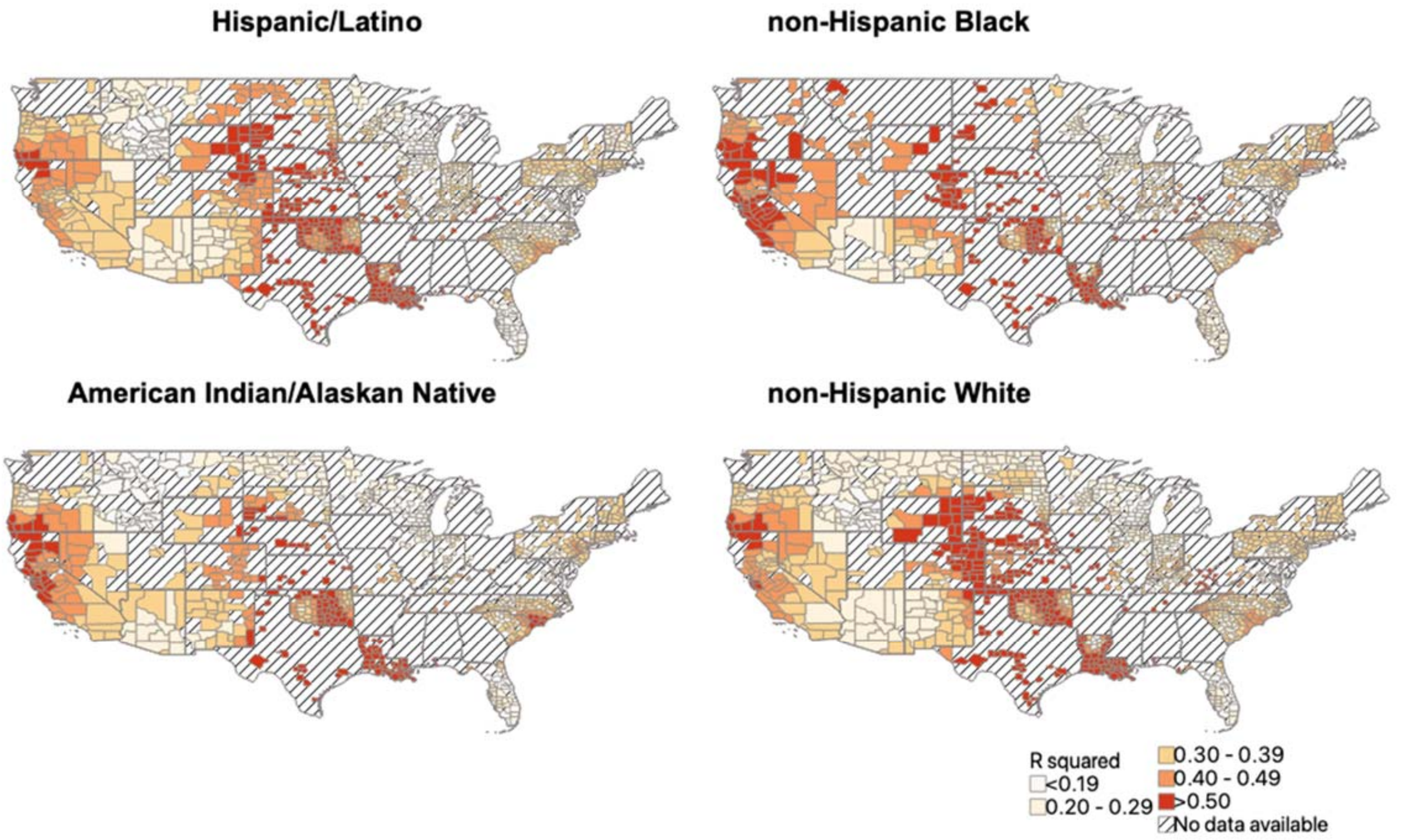

Supplementary Fig. 5**. County-level R^2 values from geographically weighted regression models assessing the association between a 10 percent higher proportion of county population categorized as Hispanic/Latino, American Indian/Alaskan Native, and non-Hispanic Black, residents and geometric mean county-level community water system (CWS) and uranium.** High R^2 values are shown in darker colors and indicate a higher goodness-of-fit. Models were adjusted for population density, the percent of public drinking water supplied from groundwater sources, median household income, and the percent of residents with a high school diploma. Counties with missing data are shown with light gray hatch marks. The number of counties included in the geographically weighted regression analysis for each racial/ethnic group were: Non-Hispanic Black (n= 1,848), American Indian/Alaskan Native (n= 1,522), Hispanic/Latino (n= 2,341).

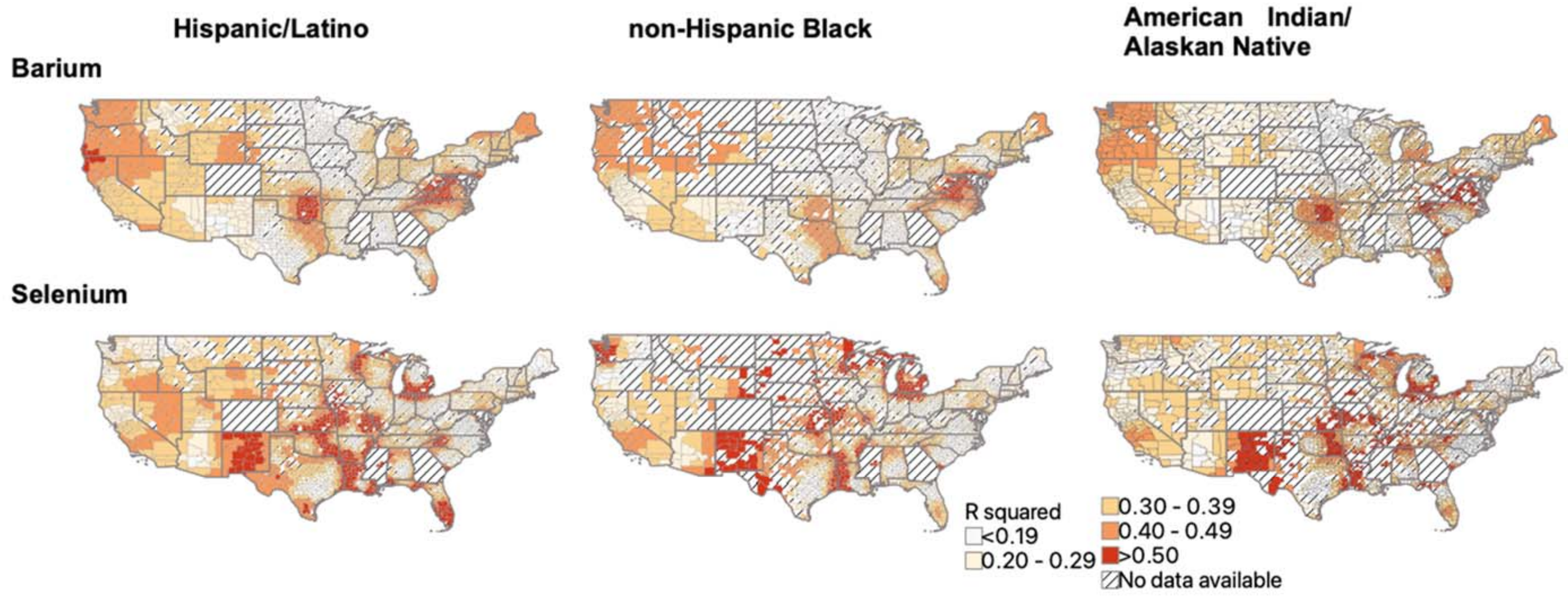

Supplementary Fig. 6**. Direction and magnitude of the geometric mean ratio (GMR) of county-level community water system (CWS) barium concentrations per 10 percent higher county proportion of non-Hispanic Black, American Indian/Alaskan Native, Hispanic/Latino, and non-Hispanic White residents from geographically weighted regression models.** Models were adjusted for population density, the percent of public drinking water supplied from groundwater sources, median household income, and the percent of residents with a high school diploma. Counties with a positive effect estimate (GMR>1) are shown in red scale colors, counties with a negative effect estimate (GMR<1) are shown in blue scale colors, and counties where a null effect estimate was observed are shown in white. Counties with missing data are shown with light gray hatch marks. The number of counties included in the geographically weighted regression analysis for each racial/ethnic group were Non-Hispanic Black (n= 1,848), American Indian/Alaskan Native (n= 1,522), Hispanic/Latino (n= 2,341), and non-Hispanic White (n = 2,555).

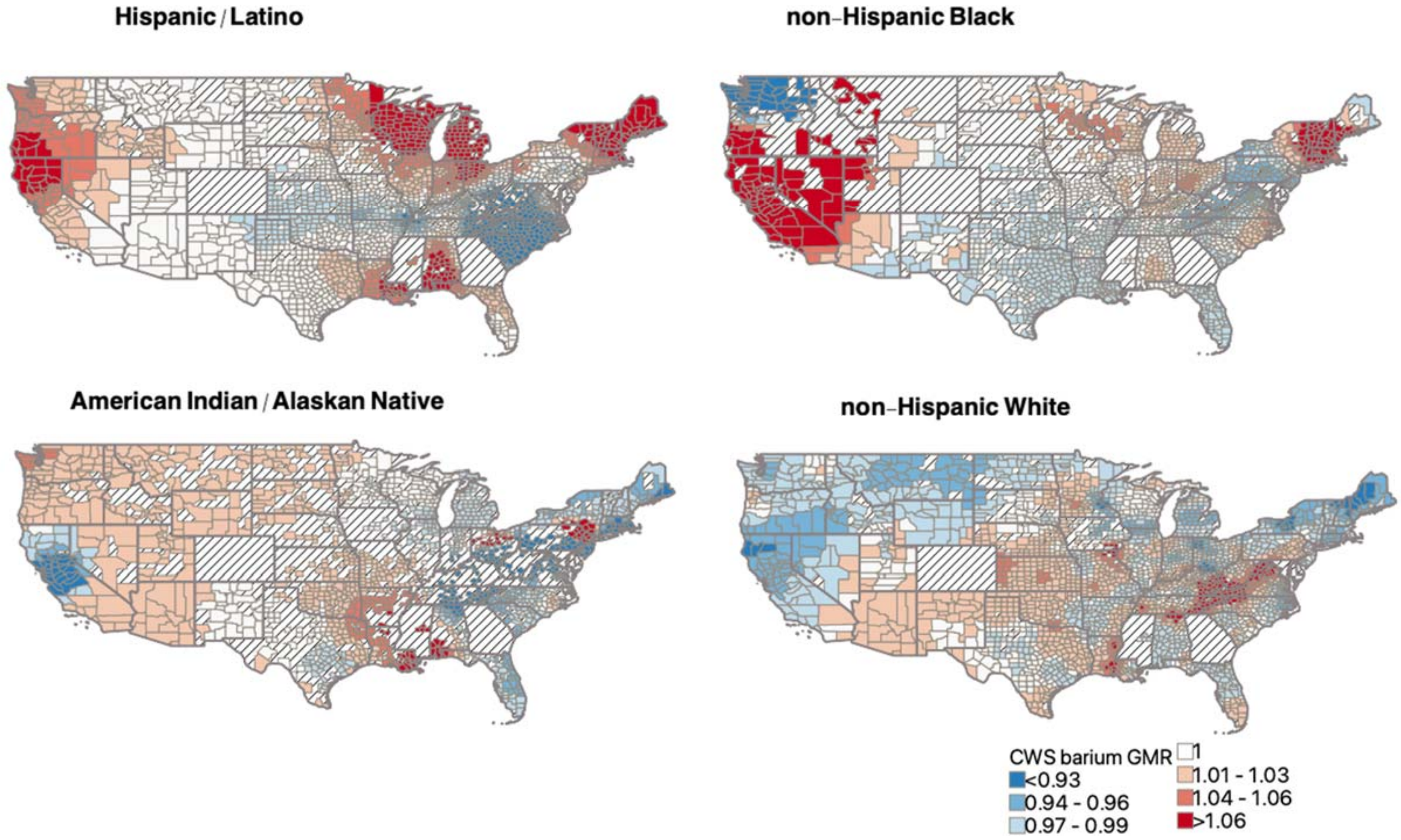

Supplementary Fig. 7. **Direction and magnitude of the geometric mean ratio (GMR) of county-level community water system (CWS) selenium concentrations per 10 percent higher county proportion of non-Hispanic Black, American Indian/Alaskan Native, Hispanic/Latino, and non-Hispanic White residents from geographically weighted regression models.** Models were adjusted for population density, the percent of public drinking water supplied from groundwater sources, median household income, and the percent of residents with a high school diploma. Counties with a positive effect estimate (GMR>1) are shown in red scale colors, counties with a negative effect estimate (GMR<1) are shown in blue scale colors, and counties where a null effect estimate was observed are shown in white. Counties with missing data are shown with light gray hatch marks. The number of counties included in the geographically weighted regression analysis for each racial/ethnic group were Non-Hispanic Black (n= 1,848), American Indian/Alaskan Native (n= 1,522), Hispanic/Latino (n= 2,341), and non-Hispanic White (n = 2,555).

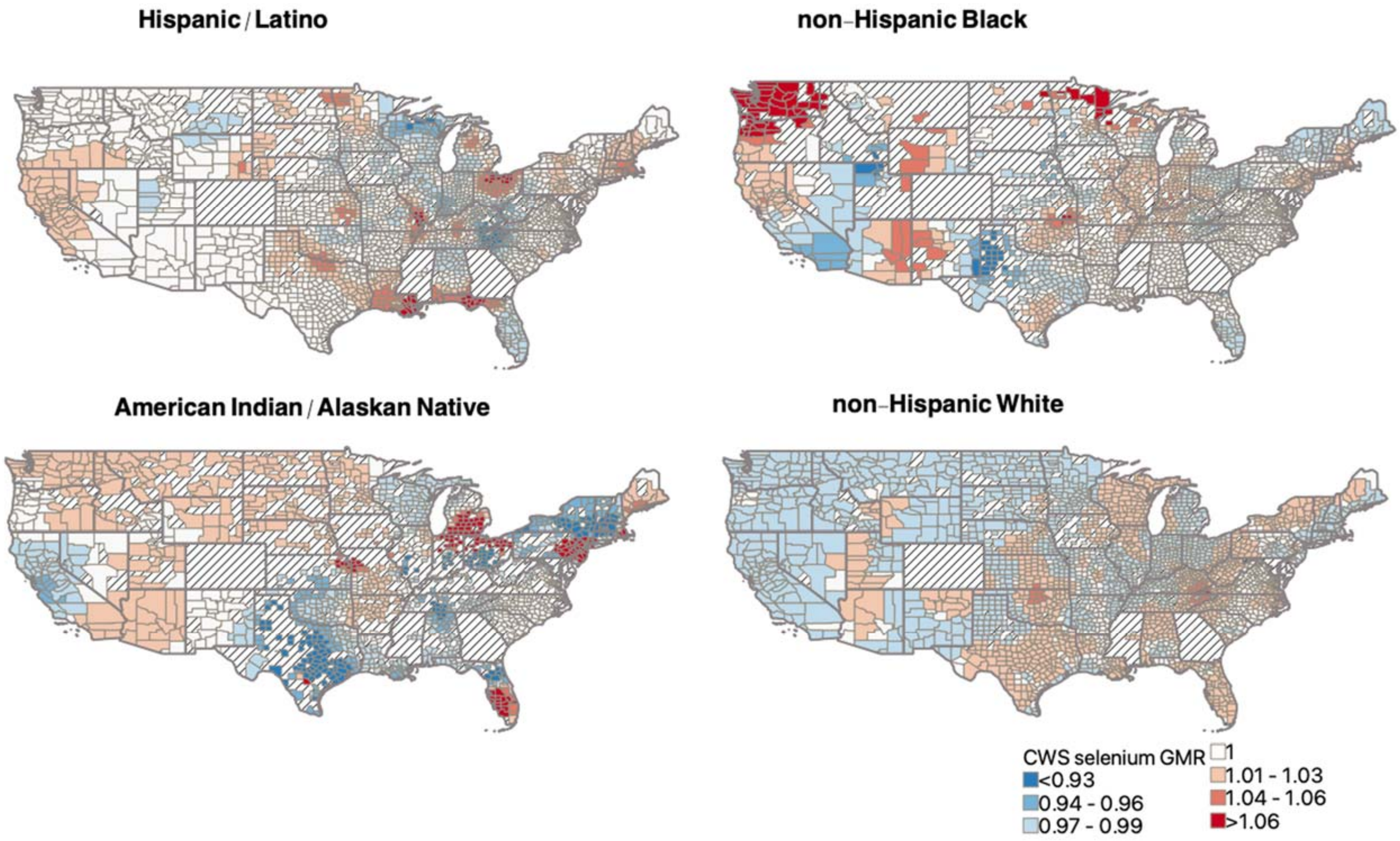

Supplementary Table 4. **County-level mean estimated community water system (CWS) metal concentrations and sociodemographic characteristics for all counties excluded from the analysis (counties with< 100 residents of each racial/ethnic group). No counties were excluded for having <100 non-Hispanic White residents.**

N	**All conterminous US counties** 3158	**<100 non-Hispanic Black residents** 757	**<100 Hispanic/Latino residents** 246	**<100 American Indian/Alaskan Native residents** 1078	**Missing CWS metal concentration estimates** 473
CWS metal estimates (mean, SD)					
Arsenic (2006-2011)	1.45 (2.3) N= 2661	2.17 (2.97) N= 737	2.04 (3.42) N= 244	1.51 (2.53) N= 1063	*NA*
Uranium (2000-2011)	3.52 (6.87) N= 1192	5.88 (10.12) N= 342	4.33 (8.3) N= 104	4.9 (9.42) N= 396	*NA*
Selenium (2006-2011)	56.74 (105.75) N= 2603	68.25 (79.82) N= 648	64.25 (70.63) N= 235	62.79 (79.11) N= 1044	*NA*
Barium (2006-2011)	1.34 (2.67) N= 2603	2.09 (3.83) N= 715	1.94 (4.74) N= 235	1.72 (3.5) N= 1044	*NA*
Sociodemographic characteristics					
Population size (mean, SD)	196007 (1195195)	10755 (8187)	5861 (4024)	15459 (12598)	707480 (2932434)
Population density (mean, SD)	264 (1728)	17 (19)	19 (77)	49 (151)	279 (944)
% public drinking water sourced from groundwater supplies (mean, SD)	63 (42)	76 (39)	72 (42)	70 (42)	68 (43)
Median household income (mean, SD)	43848 (11029)	42400 (8166)	39623 (7466)	41039 (8318)	42974 (13327)
% adults with high school diploma (mean, SD)	83 (10)	86 (10)	86 (11)	86 (10)	78 (12)
% population living in rural area (mean, SD)	58 (32)	81 (23)	95 (15)	77 (25)	58 (33)
Racial/ethnic composition (mean, SE)					
% non-Hispanic Black	9(14.5)	3.6 (3.2)	3.7 (13)	6.5 (14)	20 (21)
% American Indian/Alaskan Native	1.7 (661)	3 (10)	2.8 (10)	0.4 (0.3)	1 (6)
% Hispanic/Latino	8.4 (13)	8 (15)	1.5 (1.6)	6 (13)	6 (7)
% non-Hispanic White	78.5 (19.5)	87 (17)	90 (16)	85 (18)	70 (21)

Supplementary Table 5**. Description and source of county-level variables considered and utilized in the current analysis.**

**Variable and description**	**Source**
**County-level community water system (CWS) metal concentration estimates:**<br><br>County-level, population-weighted CWS metal concentration estimates (mean, 95th percentile) for arsenic, uranium, selenium, and barium. Arsenic, selenium, and barium estimates are for the period 2006-2011, and uranium estimates are for the period 2000-2011 to account for the different compliance monitoring periods required for uranium under the Radionuclides Rule.	Previously developed and described in detail by Nigra et al. 2020 [1] and Ravalli et al. 2022 [2]. Developed from routine compliance monitoring records collected by the US EPA for the National Contaminant Occurrence Database in support of the Second (2000-2005) and Third (2006-2011) Six Year Review. Estimates are publicly available via:<br>• https://github.com/annenigra/US-PublicWaterSystem-Metal-Estimates<br>• https://msph.shinyapps.io/drinking-water-dashboard/
**Racial/ethnic composition variables:**<br><br>County-level % non-Hispanic African American, % American Indian or Alaskan Native, % Asian, % Native Hawaiian or Other Pacific Islander, % Hispanic, % non-Hispanic White residents.	 <br>Estimates were derived from 2011 US Census Population Estimates[3]
**Percent of population living in rural areas**	Previously developed and published in the 2013 County Health Ranking database by the University of Washington Population Health Institute .<br><br>Estimates were derived from 2011 US Census Population Estimates.
**Percent of adults with a high school diploma**	Previously developed and published in the 2013 County Health Ranking database by the University of Washington Population Health Institute[4]<br><br>Estimates were derived from the 2007-2011 US Census American Community Survey.
**Median household income**	Previously developed and published in the 2013 County Health Ranking database by the University of Washington Population Health Institute [4]<br><br>Estimates were derived from the 2011 Small Area Income and Poverty Estimates and 2010-2011 National Center for Education Statistics data
**Percent of residents without health insurance**	Previously developed and published in the 2013 County Health Ranking database by the University of Washington Population Health Institute [4]<br><br>Estimates were derived from the 2010 Small Area Health Insurance Estimates.
**Percent unemployment**	Previously developed and published in the 2013 County Health Ranking database by the University of Washington Population Health Institute [4]

	Estimates were derived from 2011 Bureau of Labor Statistics data.
**Percent of children living below poverty level,** and **Percent of public school children eligible for free/reduced lunch**	Previously developed and published in the 2013 County Health Ranking database by the University of Washington Population Health Institute [4]<br><br>Estimates were derived from the 2011 Small Area Income and Poverty Estimates and 2010-2011 National Center for Education Statistics data.
**Population density**	Estimates were derived from the 2010 US Census[3].
**Percent of public drinking water supplied by groundwater sources**	Estimates were calculated by the US Geological Survey for 2010 [5]
**Social Vulnerability Index for socioeconomic status**	Extracted from the Center for Disease Control(CDC)/Agency for Toxic Substances and Disease Registry (ATSDR) 2010 and 2014 database[6]

Supplementary Table 6. **Diagnostics for spatial lag and spatial error models from ordinary least squares (OLS) regression models assessing the association between racial/ethnic composition and community water system metal concentration estimates.** The OLS model estimates for the two-sided Shapiro Wilk test to assess the normality of residuals are presented in column 2. Statistically significant values indicate a non-normal distribution of residuals. In columns 3 to 6, the estimates from the one-sided Lagrange Multiplier (LM) test for a missing spatially lagged dependent variable (lag), and for error dependence (error) , and the respective p values are presented. Statistically significant values indicate the presence of spatial dependence, a higher magnitude of the estimate indicate a better goodness of fit of the model to address spatial autocorrelation of errors. All models were adjusted for population density, the percent of public water sources from groundwater supplies, median household income, percent of adults with a high school diploma, and the racial/ethnic composition of other racial/ethnic groups, except non-Hispanic White (leave-one-out model). Models assessing increases in the proportion of non-Hispanic White residents were not adjusted for the proportion of Hispanic residents (leave-one-out model). Estimates were considered statistically significant when p value <0.01, significant p values are indicated with a * symbol next to the estimates.

	OLS Regression	Spatial Regression	
	Shapiro Wilk test	Spatial Lag	Spatial Error
Non-Hispanic African American			
Arsenic	0.9575*	7.6249*	8.1938*
Uranium	0.9569*	1.534	1.3625
Selenium	0.9003*	8.7594*	7.1913*
Barium	0.9495*	13.931*	10.218*
American Indian or Alaskan Native			
Arsenic	0.9679*	7.6249*	8.1938*
Uranium	0.9572*	1.534	1.3625
Selenium	0.9007*	8.7594*	7.1913*
Barium	0.9491*	13.931*	10.218*
Hispanic			
Arsenic	0.9679*	7.6249*	8.1938*
Uranium	0.9572*	1.534	1.3625
Selenium	0.9007*	8.7594*	7.1913
Barium	0.9491*	13.931*	10.218
non-Hispanic White			
Arsenic	0.9565*	7.5254*	7.2982*
Uranium	0.9392*	1.4026	1.2575
Selenium	0.8993*	8.7549*	6.9567*
Barium	0.9488*	13.814*	10.003*

Supplementary Fig. 8 **Photocopy safe version for main manuscript Figures 2 (a) and 3(b).** Direction and magnitude of the geometric mean ratio (GMR) of county-level community water system (CWS) arsenic (a) and uranium (b) concentrations per 10 percent higher county proportion of non-Hispanic Black, American Indian/Alaskan Native, Hispanic/Latino, and non-Hispanic White residents from geographically weighted regression models

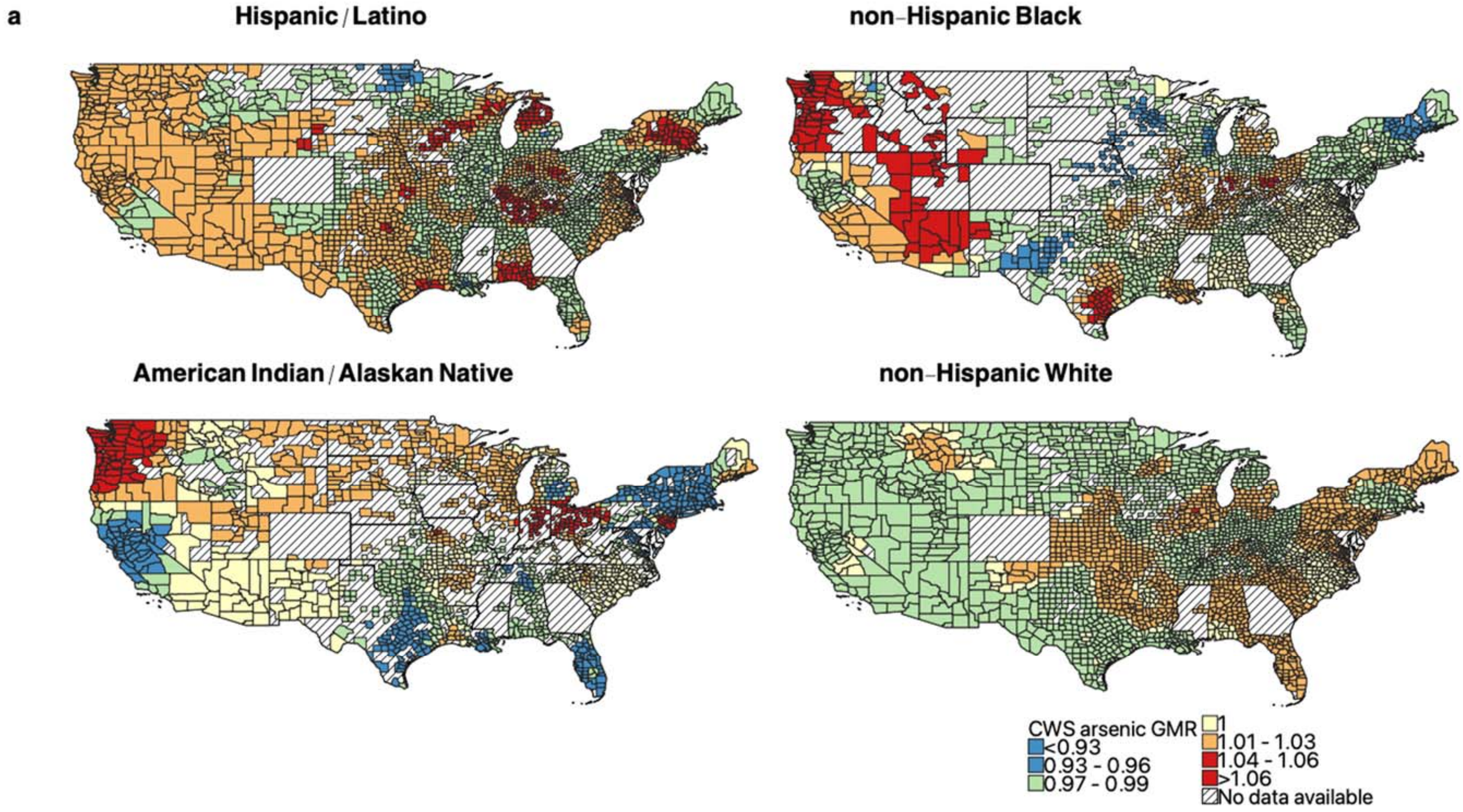

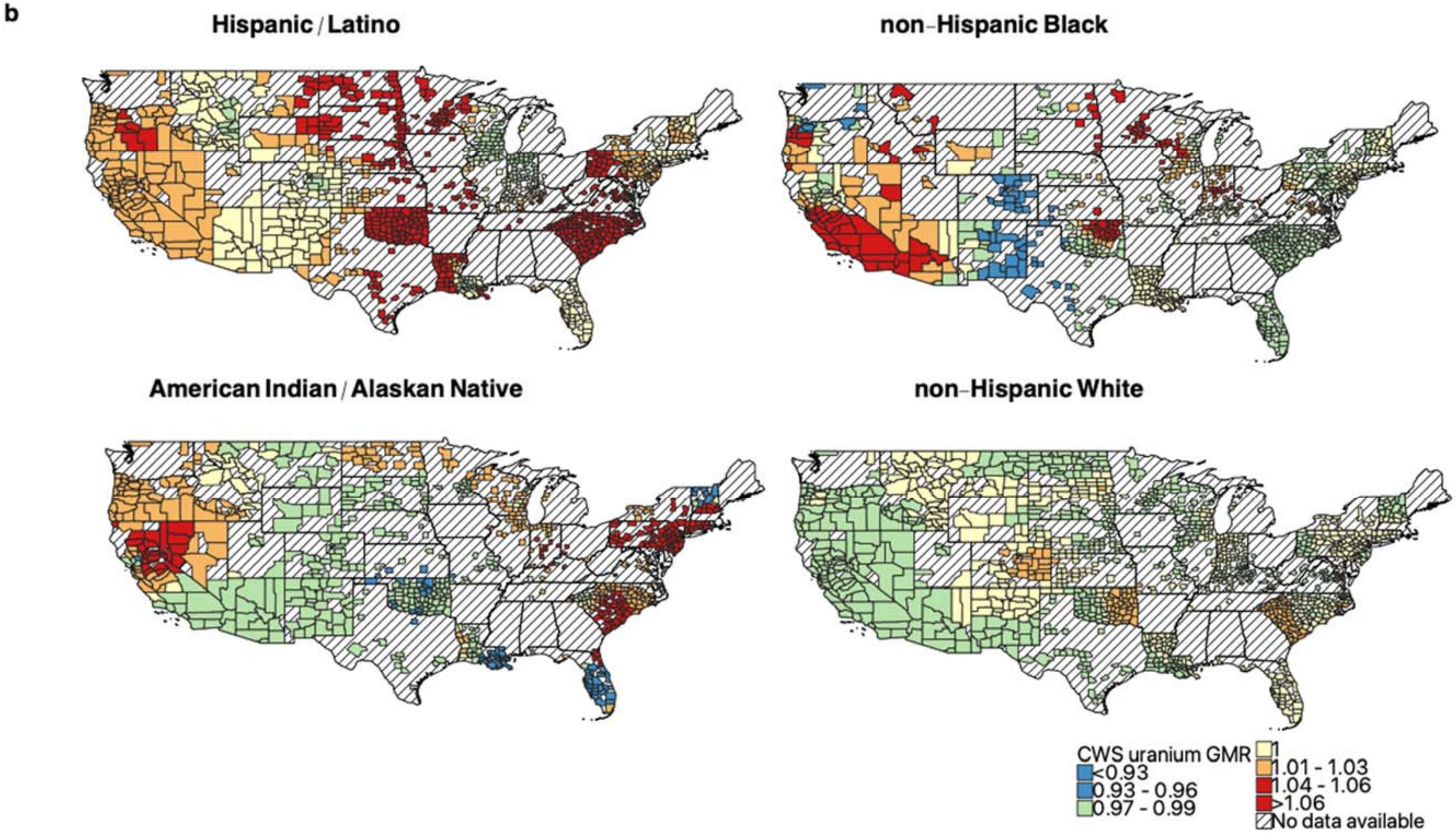
b
Hispanic / Latino
non-Hispanic Black
American Indian / Alaskan Native
non-Hispanic White
CWS uranium GMR
<0.93
0.93 - 0.96
0.97 - 0.99
1
1.01 - 1.03
1.04 - 1.06
>1.06
No data available

Supplementary References

1. Nigra, A. E. et al. Inequalities in public water arsenic concentrations in counties and community water systems across the United States, 2006–2011. *Environ. Health Perspect.* **128,** 12 (2020).

2. Ravalli, F. et al. Sociodemographic inequalities in uranium and other metals in community water systems across the US, 2006-2011. Lancet Planet. Health. **6**, E-320-E330 (2022).

3. Bureau, U. C. USA Counties: 2011. Census.gov https://www.census.gov/library/publications/2011/compendia/usa-counties-2011.html., (2011)

4. County Health Rankings & Roadmaps. Rankings Data & Documentation. National Data & Documentation: 2010-2018. https://www.countyhealthrankings.org/explore-health-rankings/rankings-data-documentation/national-data-documentation-2010-2018., (2013).

5. Maupin, M. A. et al. Estimated use of water in the United States in 2010. Estimated use of water in the United States in 2010 vol. 1405 64 http://pubs.er.usgs.gov/publication/cir1405, (2014).

6. Centers for Disease Control and Prevention/ Agency for Toxic Substances and Disease Registry/ Geospatial Research, Analysis, and Services Program. 2010 Social Vulnerability Index database. https://www.atsdr.cdc.gov/placeandhealth/svi/data_documentation_download.html, (2021).
