# Supplementary material for: Nationwide geospatial analysis of county racial and ethnic composition and public drinking water arsenic and uranium
Source: Nat Commun. 2022 Dec 3;13:7461. doi: 10.1038/s41467-022-35185-6 (PMC9718774; doi:10.1038/s41467-022-35185-6)
Supplement: Supplementary file 3 — Reporting Summary [file 41467_2022_35185_MOESM3_ESM.pdf]

nature portfolio

Corresponding author(s): Irene Martinez-Morata

Last updated by author(s): 11/11/2022

Reporting Summary

Nature Portfolio wishes to improve the reproducibility of the work that we publish. This form provides structure for consistency and transparency in reporting. For further information on Nature Portfolio policies, see our Editorial Policies and the Editorial Policy Checklist.

Statistics

For all statistical analyses, confirm that the following items are present in the figure legend, table legend, main text, or Methods section.

n/a	Confirmed	
☐	☒	The exact sample size (*n*) for each experimental group/condition, given as a discrete number and unit of measurement
☐	☒	A statement on whether measurements were taken from distinct samples or whether the same sample was measured repeatedly
☐	☒	The statistical test(s) used AND whether they are one- or two-sided<br>*Only common tests should be described solely by name; describe more complex techniques in the Methods section.*
☐	☒	A description of all covariates tested
☐	☒	A description of any assumptions or corrections, such as tests of normality and adjustment for multiple comparisons
☐	☒	A full description of the statistical parameters including central tendency (e.g. means) or other basic estimates (e.g. regression coefficient) AND variation (e.g. standard deviation) or associated estimates of uncertainty (e.g. confidence intervals)
☐	☒	For null hypothesis testing, the test statistic (e.g. *F*, *t*, *r*) with confidence intervals, effect sizes, degrees of freedom and *P* value noted<br>*Give P values as exact values whenever suitable.*
☒	☐	For Bayesian analysis, information on the choice of priors and Markov chain Monte Carlo settings
☒	☐	For hierarchical and complex designs, identification of the appropriate level for tests and full reporting of outcomes
☐	☒	Estimates of effect sizes (e.g. Cohen's *d*, Pearson's *r*), indicating how they were calculated

Our web collection on statistics for biologists contains articles on many of the points above.

Software and code

Policy information about availability of computer code

Data collection	We extracted the original data from publicly available sources accesible online and conducted the variables extraction, data preparation and merge of datasets in R version 4.0.3.
Data analysis	All data management and analysis were conducted in R version 4.0.3, except the assessment of spatial autocorrelation which was conducted in GeoDa version 1.18.0. The Lagrange (Lag) Multiplier diagnostics test and the spatial lag regression models were conducted using the R package “spatialreg”, the geographically weighted regression models were conducted using the R package "GWmodel" . For visualization, maps were generated in QGIS version 3.10.<br>Replication code is maintained in the following GitHub repository: DOI: 10.5281/zenodo.7301984

For manuscripts utilizing custom algorithms or software that are central to the research but not yet described in published literature, software must be made available to editors and reviewers. We strongly encourage code deposition in a community repository (e.g. GitHub). See the Nature Portfolio guidelines for submitting code & software for further information.

Data

Policy information about availability of data

All manuscripts must include a data availability statement. This statement should provide the following information, where applicable:

- Accession codes, unique identifiers, or web links for publicly available datasets
- A description of any restrictions on data availability
- For clinical datasets or third party data, please ensure that the statement adheres to our policy

All data used in this analysis are publicly available. The CWS metal concentration data are available at: https://github.com/annenigra/US-PublicWaterSystem-Metal-Estimates. An interactive map of county-level CWS metal concentrations is also available at: https://msph.shinyapps.io/drinking-water-dashboard/. County-level racial/ethnic composition and county-level sociodemographic data are available at: https://www.census.gov/library/publications/2011/compendia/usa-counties-2011.html and : https://www.countyhealthrankings.org/explore-health-rankings/rankings-data-documentation/national-data-documentation-2010-2018. The CDC Social Vulnerability Index data are available at: https://www.atsdr.cdc.gov/placeandhealth/svi/data_documentation_download.html. Data on the percent of public drinking water supplied by groundwater sources are available at: http://pubs.er.usgs.gov/publication/cir1405. A full description of all county-level variables used in this analysis is available in Supplementary Table 5. Documentation of the data sources and processed data is available in the following GitHub repository: DOI: 10.5281/zenodo.7301984

Human research participants

Policy information about studies involving human research participants and Sex and Gender in Research.

Reporting on sex and gender	N/A
Population characteristics	N/A
Recruitment	N/A
Ethics oversight	N/A

Note that full information on the approval of the study protocol must also be provided in the manuscript.

Field-specific reporting

Please select the one below that is the best fit for your research. If you are not sure, read the appropriate sections before making your selection.

☐ Life sciences ☐ Behavioural & social sciences ☒ Ecological, evolutionary & environmental sciences

For a reference copy of the document with all sections, see nature.com/documents/nr-reporting-summary-flat.pdf

Ecological, evolutionary & environmental sciences study design

All studies must disclose on these points even when the disclosure is negative.

Study description	This is a quantitative cross-sectional study that analyses the association between county-level racial?ehtnic population composition and county-level drinking water metal concentrations.
Research sample	Our research sample is the county-level population data directly obtained from the US Census estimates (publicly available), which is representative of the US population. We chose this sample as the study population because the US census collected information about several sociodemographic characteristics, including racial/ethnic composition nationwide, and provides estimates at the county level, which matches the organization level of the county-level water contaminants data that our team previously developed.
Sampling strategy	We included all available population estimates from the US Census data for all contermious counties in the US.
Data collection	The data was collected from publicly available sources accesible online, the variables selection and data merge and preparation was conducted in R version 4.0.3. All data used in this analysis are publicly available. The CWS metal concentration data was developed from EPA monitoring records previously published and are available at: https://github.com/annenigra/US-PublicWaterSystem-Metal-Estimates. We downloaded the county-level racial/ethnic composition and county-level sociodemographic data from the US Census using and the County Health Ranking database websites. The CDC Social Vulnerability Index was extracted from the CDC website, data on the percent of public drinking water supplied by groundwater sources was extracted from the US Geological Survey.
Timing and spatial scale	We utilized previously developed, county-level, population-weighted concentration estimates of arsenic, uranium, selenium, and barium concentrations in community water systems (CWSs) across the US. CWS metal concentrations were averaged within a six-year period (2006-2011) for arsenic, selenium, and barium and within an eleven-year period (2000-2011) for uranium. County-level racial/ethnic composition variables were derived from 2010 US Census Population Estimates. The water metal estimates are the most recently available estimates, the timing of the racial/ethnic compositon and sociodemographic variables was selected to overlap with

the timing of the water estimates.

Data exclusions	We excluded counties with no data on community water system metal concentrations.
Reproducibility	Replication code is maintained in the following GitHub repository: DOI: 10.5281/zenodo.7301984
Randomization	No randomization was conducted. We adjusted our models for potential confounders incuding: population density, the percent of public drinking water supplied from groundwater sources, median household income, and the percent of residents with a high school diploma.
Blinding	Blinding was not relevant for the study objectives. This is an observational study conducted with publicly available data.

Did the study involve field work? ☐ Yes ☒ No

Reporting for specific materials, systems and methods

We require information from authors about some types of materials, experimental systems and methods used in many studies. Here, indicate whether each material, system or method listed is relevant to your study. If you are not sure if a list item applies to your research, read the appropriate section before selecting a response.

Materials & experimental systems

n/a	Involved in the study
☒	☐ Antibodies
☒	☐ Eukaryotic cell lines
☒	☐ Palaeontology and archaeology
☒	☐ Animals and other organisms
☒	☐ Clinical data
☒	☐ Dual use research of concern

Methods

n/a	Involved in the study
☒	☐ ChIP-seq
☒	☐ Flow cytometry
☒	☐ MRI-based neuroimaging
